# Symbiotic Efficiency of Native Rhizobia Nodulating Common Bean (*Phaseolus vulgaris L.*) in Soils of Western Kenya

**DOI:** 10.1155/2014/258497

**Published:** 2014-11-10

**Authors:** Fanuel Kawaka, Mathews M. Dida, Peter A. Opala, Omwoyo Ombori, John Maingi, Newton Osoro, Morris Muthini, Alice Amoding, Dative Mukaminega, John Muoma

**Affiliations:** ^1^Department of Applied Plant Sciences, Maseno University, Maseno, Kenya; ^2^Department of Pure and Applied Sciences, Technical University of Mombasa, P.O. Box 90420, Kenya; ^3^Department of Soil Science, Maseno University, Maseno, Kenya; ^4^Department of Plant Sciences, Kenyatta University, P.O. Box 43844-00100, Nairobi, Kenya; ^5^Department of Microbiology, Kenyatta University, P.O. Box 43844-00100, Nairobi, Kenya; ^6^Department of Biochemistry and Biotechnology, Kenyatta University, P.O. Box 43844-00100, Nairobi, Kenya; ^7^Department of Soil Science, Makerere University, P.O. Box 7062, Kampala, Uganda; ^8^Faculty of Applied Sciences, Kigali Institute of Science and Technology, P.O. Box 3900, Kigali, Rwanda; ^9^Department of Biological Sciences, Masinde Muliro University of Science and Technology, P.O. Box 190-50100, Kenya

## Abstract

This study was conducted to determine the abundance and symbiotic efficiency of native rhizobia nodulating common bean in Kisumu and Kakamega, Kenya. Soil sampling was carried out in three farms that had been used for growing common bean for at least two seasons and one fallow land with no known history of growing common bean or inoculation. Abundance of soil rhizobia and symbiotic efficiency (SE) were determined in a greenhouse experiment. Native rhizobia populations ranged from 3.2 × 10^1^ to 3.5 × 10^4^ cells per gram of soil. Pure bacterial cultures isolated from fresh and healthy root nodules exhibited typical characteristics of *Rhizobium* sp. on yeast extract mannitol agar media supplemented with Congo red. Bean inoculation with the isolates significantly (*p* < 0.05) increased the shoot dry weight and nitrogen (N) concentration and content. The SE of all the native rhizobia were higher when compared to a reference strain, CIAT 899 (67%), and ranged from 74% to 170%. Four isolates had SE above a second reference strain, Strain 446 (110%). Our results demonstrate the presence of native rhizobia that are potentially superior to the commercial inoculants. These can be exploited to enhance bean inoculation programmes in the region.

## 1. Introduction

Many countries in sub-Saharan Africa, including Kenya, are characterized by increased population growth and food insecurity, as well as increasing environmental and economic declines associated with conventional farming. These problems have necessitated the development and adoption of alternative food production practices. Sustainable soil fertility management is key to food production and environmental stability [1]. Historically, legume crops such as common bean (*Phaseolus vulgaris L*.) have played an important role in soil fertility through symbiotic fixation of nitrogen (BFN), enhancement of soil organic matter, and prevention of nutrient leaching [2, 3]. In Kenya, common bean is ranked among the most important major grain food crops [4] and produced mainly by smallholder farmers with farms ranging from 1 to 10 ha in size.

Integration of common bean into existing cropping systems has the potential to mitigate food insecurity in Kenya if challenges associated with its production are addressed. The national annual dry bean production is about 380,000 metric tons (*t*) which is far below the pulse demand of 749,000* t* [5]. Dry bean production is predominant by smallholder farmers and has been on the decline in recent years. The low bean yields have been attributed to various constraints such as diseases, insect pests, and poor agronomic practices [6]. In addition, soil fertility depletion is a major constraint to crop production among most smallholder farmers in Kenya and is considered the fundamental biophysical root cause of declining per capita food production in the region [7]. In particular, deficiency of N, the nutrient taken up by beans in the largest amounts among the essential plant nutrients [8], is a major constraint to crop productivity on many smallholder farming systems [9]. Unfortunately, smallholder farmers, who are the major dry bean producers in Kenya, rarely apply nitrogenous fertilizers in bean production, relying mainly on the ability of the bean to fix its own nitrogen and natural soil fertility, despite the fact that beans are known to be poor nitrogen-fixers [10]. Inoculation of legumes is necessary in the absence of compatible rhizobia and when rhizobial populations are low or inefficient in fixing N [11]. Commercial inoculants used in western Kenya still contain exotic cultures from United States of America [12] which may not be well adapted to local conditions. It is therefore hypothesized that there are rhizobia strains in soils of western Kenya with better SE compared to commercial inoculants. The objective of this study was therefore to determine the abundance and effectiveness of native rhizobia nodulating common bean in western Kenya.

## 2. Materials and Methods

### 2.1. Study Site

The study was carried out in Korando B, Kisumu district, Kenya (S 00° 05.167′, E 034° 41.613′), and Masinde Muliro University of Science and Technology (MMUST), Kakamega, Kenya (N 00° 17.104′, E 034° 45.874′). The sites were selected based on agroclimatic conditions and prevalence of common bean cultivation. MMUST is located at an altitude of 1585 metres above sea level within a high potential agroecological zone and has an annual rainfall of 1200–2100 mm while Korando B is located at an altitude of 1300 meters above sea level and has an annual rainfall of 1200–1300 mm. Soils in MMUST and Kisumu are generally classified as Nitisols and Arenosols, respectively [13].

### 2.2. Determination of the Abundance of Indigenous Rhizobia in the Soil

Soil sampling was carried out in three farms with at least two seasons of growing common bean and one fallow farm with no history of common bean cultivation. The cropping systems in the farms consisted of bean, maize, maize-bean intercrop, napier, and fallow land. Using a 2.5 cm diameter soil probe, 20 soil cores were randomly collected from each farm to a depth of 15 cm and thoroughly mixed into a composite sample. Each sample was aseptically collected to avoid cross contamination between soils from different sampling points. The samples were then divided into two parts; one part was for the determination of rhizobia population in the soil while the other part was for the chemical and physical analyses. Soil samples were air-dried and passed through a 2 mm sieve for chemical analysis.

Soil pH was determined using a glass electrode pH meter in 1 : 2.5 soil : water suspension [14]. Exchangeable calcium (Ca) and magnesium (Mg) were determined by atomic absorption spectrophotometry, whereas K and Na were extracted using ammonium acetate and analyzed by flame photometry [15]. Cation exchange capacity (CEC) was determined by ammonium acetate saturation method at pH 7.0. Available P was extracted using the Bray 1 method and determined by ascorbic acid-molybdate blue colour method. Organic carbon (OC) was determined by Walkley-Black wet combustion method [16], while total N was determined by Kjeldahl method [17].

Abundance of rhizobia was determined using the most probable number (MPN) technique. Common bean (*Mwitemania* variety) was used as the trapping host. The bean seeds were selected for uniformity in size, shape, colour, and the pregermination time of seeds to ensure synchronized germinations [18]. After pretesting, seeds were dipped in 95% alcohol to remove waxy material on the surface and trapped air and then surface sterilized in 3% sodium hypochlorite solution for 3–5 minutes, followed by rinsing in 6 changes of sterile distilled water. The seeds were then soaked in water in a refrigerator (4°C) for four hours to imbibe water and then placed in a germination chamber for pregermination.

After germination, two to three healthy seedlings were aseptically transplanted into each Leonard jar filled with sterilized vermiculite. After 7 days, seedlings were thinned to 1 plant per jar and then inoculated with the diluted soil samples [18]. Soil inocula were prepared by suspending 10 g of soil sample in 90 mL of sterile water in a 160-mL dilution bottle and shaken for 20 min in a wrist-action shaker at room temperature (25°C). One mL of each suspension was aseptically pipetted into 9 mL sterile water diluent in McCartney bottle and shaken for 2 min. The resulting suspension was serially diluted tenfold from 10^−1^ to 10^−6^ with four replications at each dilution level. An aliquot of 1 mL of diluent was then used to inoculate each seedling in the Leonard jars [19]. The seedlings were irrigated with sterile nitrogen-free plant nutrient solution [20]. The nutrient consisted of 5 stock solutions containing in g/L 0.1 CaCl_2_, 0.12 MgSO_4_·7H_2_O, 0.1 KH_2_PO_4_, 0.15 Na_2_HPO_4_·2H_2_O, 0.005 ferric citrate, and 1.0 mL of trace elements stock solution. The trace elements stock solution contained 2.86 H_3_BO_3_, 2.03 MnSO_4_·7H_2_O, 0.22 ZnSO_4_·7H_2_O, 0.08 CuSO_4_·5H_2_O, and 0.14 NaMoO_2_·2H_2_O in g/L. The pH of the solution was adjusted to 6.8 using NaOH (1.0 M) or HCL (1.0 M). All solutions were sterilized by autoclaving at 121°C for 15 minutes. Regular checking of levels of nitrogen-free nutrient solution was carried out daily to ensure that the seedlings were adequately moistened. The plants in the Leonard jars were scored for the presence or absence of nodules on the 28th day after inoculation. The presence of a single nodule in a Leonard jar was considered a positive score. The MPN technique was used to determine rhizobia cells per gram of dry soil [21].

### 2.3. Nodule Sampling and Isolation of Rhizobia

Nodules were sampled during the late flowering and early pod setting stages. A total of 100 representative flowering bean plants were carefully uprooted from farms in Kisumu and MMUST after 7 weeks of emergence. After carefully washing the nodules, fresh and red nodules were carefully removed from the roots and wrapped in sterilized absorbent paper. The nodules were immersed in sterilized distilled water and let to imbibe water for one hour. Nodules were surface-sterilized in 1% NaOCl for 6 min, rinsed in several changes of sterile water, and then crushed with a flame-sterilized blunt-tipped pair of forceps. A loopful of the crushed nodule suspension was streaked across the surface of Petri dish containing yeast extract mannitol agar (YEMA) media containing Congo red and incubated in the dark at 26°C [22]. After three days, single colonies were marked and checked for purity by repeated streaking on YEMA medium and verifying a single type of colony morphology, absorption of Congo red (0.00125 mg kg^−1^), and a uniform Gram-stain reaction. Colony morphology such as color, mucosity, margin, transparency, elevation, and acid/alkaline reaction was evaluated on YEMA containing bromothymol blue (BTB) (0.00125 mg kg^−1^) as indicator. The pure rhizobial isolates were coded as* KSM001, KSM002, KSM003, KSM004, KSM005, KSM006, KSM007,* and* KSM008* from Kisumu and* MMUST001, MMUST002, MMUST003, MMUST004, MMUST005,* and* MMUST006* from MMUST. All the isolates were incubated at 28°C and stored at −20°C in 25% glycerol-YEM broth.

### 2.4. Authentication and Determination of Symbiotic Efficiency

Each of the pure isolates was authenticated as root nodulating bacteria by reinoculating 1 mL of three-day-old pure YEM broth culture of the isolate on the host plant grown in a controlled environment in sterilized vermiculite in Leonard jar [23]. The jars were arranged in randomized complete block design (RCBD) with four replications. The plants were watered with nitrogen-free nutrient solution. Treatments without inoculation and chemical nitrogen fertilizer served as negative control while treatments without inoculation plus nitrogen fertilizer at a rate of 70 *μ*g N mL^−1^ applied as KNO_3_ solution were used as positive control. The isolates were also compared with commercial rhizobia Strain 446 and CIAT 899 as reference strains. After 45 days, shoot dry weight (SDW), number of nodules (NN), and nodules dry weight (NDW) were measured. SDW and NDW were determined from material dried at 70°C for 24 hours [24]. Tissue N concentration per plant was analyzed using the Kjeldahl method [25] and the N content per plant calculated by multiplying the SDW with the tissue N concentration. Symbiotic efficiency (SE) was calculated by comparing each of the native isolates with N applied control (plant N content in inoculated pots/plant N content in N application) × 100 [20].

### 2.5. Data Analysis

All data on root dry weight (RDW), SDW, tissue N concentration content per plant, and SE were subjected to analysis of variance (ANOVA) using General Linear Models Procedure of SAS software version 9.1. [26] and means tested for significance using the least significance difference of means (LSD) at *p* < 0.05. Pearson correlation coefficients were calculated to establish the associative relations among the agronomic traits of the test crop.

## 3. Results

### 3.1. Soil Properties and Abundance of Indigenous Rhizobia

The soil chemical properties varied across farms at the two study sites (Table 1). The soil pH was acidic and ranged from 4.98 to 6.1 in bean farms. Maize and napier farms with low rhizobial populations of 7.8 × 10^1^ and 3.8 × 10^1^ cells per g of soil had higher levels of N in the soil. Soils from farms in Kisumu were characterized by low aluminum (Al) and copper (Cu) but had high populations of native rhizobia than MMUST farms. Available P ranged from 12 ppm to 62 ppm and 6 ppm to 11.8 ppm in cultivated farms and uncultivated land, respectively. Soil texture was classified as clay and sandy loam for MMUST and Kisumu, respectively.

### 3.2. Morphology and Cultural Characteristics

Initial screening of the nodule bacteria on YEMA media with Congo red showed that the pure samples were Gram-negative with convex elevation. They failed to absorb Congo red or absorbed it lightly. On streaking on the YEMA-BTB media, the isolates acidified and turned the green media to moderately yellow and deep yellow color after 3 days of incubation in the dark. The colony diameter ranged between 1.0 mm and 5.7 mm. The colonies appeared either dull or shiny with entire margin and convex elevation on YMA plain media. The colony transparency was either opaque or translucent with firm, dry, or smooth viscous texture. The isolates were placed in 12 groups based on the differences on morphocultural characteristics (Table 2).

### 3.3. Authentication and Symbiotic Efficiency

**Figure 1:**
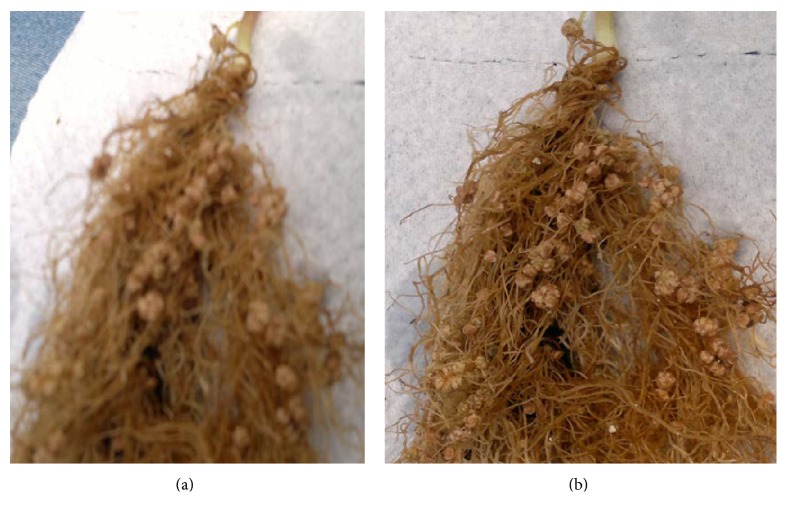
Sample nodules formed by isolates from Kisumu (a) and MMUST (b).

Upon reinoculation of the host plant, all the isolates from Kisumu and four from MMUST initiated nodulation (Figure 1) and were hence confirmed as root-nodule bacteria. Inoculation of the bean plant with different rhizobia isolates increased SDW, N concentration, and N content (Table 3). Significant differences (*p* < 0.05) were observed on RDW, N concentration, N content, and SE among rhizobia isolates tested. SE values obtained with the rhizobia isolates from the two sites were high when compared to CIAT 899. However SE values of* KSM002, KSM004, KSM006, KSM007, KSM008,* and* MMUST006* were lower than the nitrogen supplemented control plants (Tables 3 and 4). Low RDW and SDW were recorded in plants inoculated with CIAT 899 compared to N free and supplemented controls. Four of the isolates (*KSM001, KSM003, KSM005,* and* MMUST005*) tested improved the growth of beans compared to two commercial strains, CIAT 899 and Strain 446. RDW was positively correlated with SDW and N concentration (*r* = 0.245, *p* < 0.05) while SDW was strongly correlated with N content and SE (*r* = 0.545, *p* < 0.01) (Table 5).

## 4. Discussion

The pH of soils did not vary so much across the study sites regardless of the land use system. Variation observed in rhizobia population in soils among the different farms could be due to the acidic pH. Lower pH increases the solubility of Al, Mn, and Fe in soil causing toxicity to plants in excess by slowing or stopping of root growth. Niste et al. [27] reported that legumes require a neutral or slightly acidic soil for growth, especially when they depend on symbiotic N_2_ fixation. Different strains of rhizobia vary widely in certain cases in their pH tolerance. The populations of rhizobia in some of the farms were higher compared to those reported in parts of central Kenya [25]. In MMUST, the levels of soil N in maize and napier farms were above the critical values described by Okalebo et al. [28] and could have resulted in suppressing nodulation [29, 30]. Other studies found that mineral N inhibits nodule formation and functioning on root colonization of N fixing legumes [31]. High rhizobial populations in low Al and Cu soils in this study could be due to their reduced inhibitory and deleterious effects. Arora et al. [32] reported that Al and Cu are extremely toxic to growth and enzyme activity of both fast and slow-growing rhizobial species. High rhizobia populations present in the soils from the farms in Kisumu may be as a result of the light sandy loam texture of the soil. In Polish soils, light-textured soils were found to be beneficial for the proliferation and survival of root-nodule bacteria [33]. In this study, the results show the presence of native rhizobia that nodulate common bean in fallow soils with no history of growing common bean or inoculation. Anyango et al. [34] similarly demonstrated the occurrence of rhizobia indigenous to African soils with no history of deliberate inoculation that can nodulate and fix nitrogen with* P. vulgaris*. The high values of P in the cultivated farms compared to fallow land may have resulted from continuous farm organic amendment and external P fertilization. The levels of organic carbon and total N in the soils from the two sites are above the critical values described by Okalebo et al. [28] and can be considered as moderately fertile.

Growth of the isolates on YEMA media confirmed morphological characteristics of* Rhizobium *sp. [23]. Gram-negative reaction and absorption of Congo red dye during incubation of the isolates in the dark are typical characteristics of rhizobia nodulating common bean [35]. Turning of YEMA-BTB media into moderately yellow to deep yellow color is a characteristic feature of acid producing and fast growing rhizobia [36]. This is contrary to other studies in Kenya and some parts of southern Ethiopia where both slow and fast growing rhizobia have been isolated and identified [37, 38]. The colony diameter lying between 1 mm and 5.7 mm with regular and circular margins is consistent with other studies [39, 40]. The production of exopolysaccharides (EPS) by the isolates could be an adaptive feature in providing protection to bacteria against factors like temperature, salinity, and pH fluctuations in the soil. Rhizobia with ability to withstand such environmental stresses could be suitable for the development of commercial inoculants. These initial tests have been previously used to separate impurities and contaminants from pure rhizobia isolates [41].

The development of legume inocula requires that the rhizobia must be highly effective in fixing nitrogen. At final harvest, inoculated beans had higher SDW compared to the control indicating that inoculation with native isolates improved the growth of plants and are therefore efficient in N fixation. These results are contrary to those described by Mungai and Karubiu [42], who reported that inoculation with native rhizobia isolated from two farms in Njoro, Kenya, did not improve RDW or SDW of common bean. The superior ability of the isolated native rhizobia strains in this study compared to CIAT 899 and Strain 446 that are commonly recommended as inoculant strains for common bean could have been due to the presence of more effective strains of native rhizobia in the sampled farms. The frequent occurrence of* Rhizobium *isolates more effective in N_2_ fixation compared to CIAT 899 has been reported in East and Southern African soils [34], indicating the potential benefits of isolates from natural environments. Based on the differences in SDW of inoculated and nitrogen-fertilized plants as a measure of symbiotic efficiency [43], 42% of the isolates were effective N fixers and 16% performed as good as the positive controls. The high correlation of SDW with most of the tested parameters confirmed its reliability as an indicator for efficiency in N fixation.

## 5. Conclusions

This study has demonstrated the presence of native rhizobia that are potentially superior to the commercial inoculants and can be exploited to enhance bean inoculation programmes in the area. The identity of native rhizobia that improved the growth of common bean plants should be established and subjected to further greenhouse and field trials to ascertain their stability under different environmental conditions.

## Figures and Tables

**Table 1:** Native rhizobia population and physicochemical characteristics of soils from the study farms in Kisumu and MMUST.

Soil properties	MMUST farms				KISUMU farms			
	Maize	Fallow	Bean	Napier	Bean	Maize	Fallow	Maize-bean
MPN (no. rhizobia in gm^−1^ of soil)	7.8 × 10^1^	1.98 × 10^2^	4.102 × 10^3^	3.9 × 10^1^	1.25 × 10^4^	3.5 × 10^4^	3.2 × 10^1^	1.25 × 10^4^
pH (1 : 2.5 soil water ratio)	5.12	5.01	4.98	5.4	6.1	6.06	5.98	5.24
EC (dS/m)	0.3	0.7	0.7	0.4	0.2	0.2	0.4	0.4
Total *N* (%)	0.35	0.18	0.24	0.31	0.11	0.13	0.15	0.2
Organic carbon (%)	2.6	1.58	2.66	2.8	1.32	1.15	1.78	1.39
K (cmol/kg)	1.75	0.75	0.93	1.2	1.39	1.42	1.39	1.42
Na (cmol/kg)	0.6	0.5	0.7	0.8	0.6	0.7	0.6	0.8
Mg (cmol/kg)	1.19	1.01	1.39	1.53	1.17	1.4	1.08	1.77
Ca (cmol/kg)	2.74	2.84	3.08	3.7	3.02	2.66	2.56	5.31
Al (cmol/kg)	2.8	1.9	2.6	2.5	0.6	0.7	0.6	0.9
Zn (ppm)	7.2	4.5	7.6	20	9.8	6	6.2	1.7
Cu (ppm)	5.6	5.4	5.1	5.6	1.6	1.8	1.4	1.6
Fe (ppm)	16.1	13.3	10.1	24.6	22	12.5	20.4	43.2
Mn (ppm)	72.4	43.3	75.2	91.1	38.3	48.6	32.3	94.1
P (ppm)	24	6	28	35	35	62	11.8	12
Soil texture	Clay	Clay	Clay	Clay	SL	SL	SL	SL

Note: SL: sandy loam.

**Table 2:** Morphological and cultural characteristics of the rhizobia isolates from Kisumu and MMUST.

Characteristics	Isolates											
	*KSM001 *	*KSM002 *	*KSM003 *	*KSM004 *	*KSM005 *	*KSM006 *	*KSM007 *	*KSM008 *	*MMUST003 *	*MMUST004 *	*MMUST005 *	*MMUST006 *
Congo red absorption	✓	✓	✓	✓	✓	✓	✓	✓	✓	✓	✓	✓
BTB reaction	✓	✓	✓	✓	✓	✓	✓	✓	✓	✓	✓	✓
Colony colour	Cream yellow	Cream white	Cream white	Milky white	Milky white	Cream yellow	Cream white	Cream white	Milky white	Cream yellow	Milky white	Cream yellow
Colony transparency	Opaque	Translucent	Opaque	Opaque	Translucent	Translucent	Opaque	Translucent	Opaque	Translucent	Opaque	Opaque
Colony appearance	Shiny	Shiny	Shiny	Dull	Dull	Shiny	Shiny	Shiny	Shiny	Shiny	Dull	Dull
EPS production	✓	✓	✓	x	x	✓	✓	✓	x	✓	x	✓
Colony texture	Firm, dry	Smooth, viscous	Smooth, viscous	Firm, dry	Firm, dry	Smooth, viscous	Smooth, viscous	Smooth, viscous	Smooth, viscous	Smooth, viscous	Firm, dry	Firm, dry
Colony shape	Circular	Oval	Oval	Circular	Circular	Circular	Oval	Oval	Circular	Circular	Circular	Circular
Colony elevation	Convex	Convex	Convex	Convex	Convex	Convex	Convex	Convex	Convex	Convex	Convex	Convex
Colony diameter (mm)	3.7	4.7	5.7	3.7	4.0	3.7	5.0	3.3	4.7	3.3	3.0	1.0
Gram stain	✓	✓	✓	✓	✓	✓	✓	✓	✓	✓	✓	✓
Colony margin	Entire	Entire	Entire	Entire	Entire	Entire	Entire	Entire	Entire	Entire	Entire	Entire

BTB: bromothymol blue, EPS: exopolysaccharides, ✓: positive reaction, and x: negative.

**Table 3:** Effect of isolate inoculation on root dry weight, shoot dry weight, N concentration, N content, and SE under controlled experiments in Korando B, Kenya.

Isolate	RDW (g plant^−1^)	SDW (g plant^−1^)	N concentration (mg plant^−1^)	N content (mg plant^−1^)	Symbiotic efficiency (%)
*KSM001 *	0.90ab	1.59bc	1.82ab	2.89abc	125.0abc
*KSM002 *	0.94ab	1.52c	1.50abc	2.31bc	100.0bc
*KSM003 *	0.96ab	1.84bc	2.06a	3.94a	170.0a
*KSM004 *	0.82b	1.64bc	1.22c	2.07bcd	89.0bcd
*KSM005 *	1.03ab	2.01a	1.45bc	3.01ab	130.0ab
*KSM006 *	0.86ab	1.65ab	1.04cd	1.72bcd	74.0bcd
*KSM007 *	0.86ab	1.65bc	1.31bc	2.17bcd	94.0bcd
*KSM008 *	0.90ab	1.88ab	1.01cd	1.89bcd	81.0bcd
Strain 446	1.07a	1.69abc	1.51abc	2.56abc	110.0abc
CIAT 899	0.56c	1.50c	1.08c	1.55cd	67.0cd
PCNTL	1.07a	1.67abc	1.37bc	2.32bc	100.0bc
NCTL	1.00ab	1.63bc	0.46d	0.75d	—
LSD	0.22	0.36	0.59	1.43	62.0

NCTL: negative control; PCNTL: positive control; LSD is the least significant difference of means; Strain 446 and CIAT 899 are reference commercial inoculants. Means within a column followed by the same letter(s) are not significantly different at *p* < 0.05.

**Table 4:** Effect of isolate inoculation on root dry weight, shoot dry weight, N concentration, N content, and SE under controlled experiments in MMUST, Kenya.

Isolate	RDW (g plant^−1^)	SDW (g plant^−1^)	N concentration (mg plant^−1^)	N content (mg plant^−1^)	Symbiotic efficiency (%)
*MUST003 *	0.89ab	1.76a	1.32b	2.33b	100.0b
*MUST004 *	0.80b	1.78a	1.41b	2.48b	107.0b
*MUST005 *	1.08a	1.85a	2.02a	3.80a	164.0a
*MUST006 *	1.07a	1.73a	1.06b	1.80bc	78.0bc
Strain 446	1.07a	1.69a	1.51ab	2.56b	110.0b
CIAT 899	0.56c	1.50a	1.08b	1.55bc	67.0bc
PCNTL	1.07a	1.68a	1.37b	2.32b	100.0b
NCTL	1.00ab	1.63a	0.46c	0.75c	—
LSD (5%)	0.24	0.39	0.56	1.13	49.0

NCTL: negative control; PCNTL: positive control; LSD is the least significant difference of means; Strain 446 and CIAT 899 are reference commercial inoculants; means within a column followed by the same letter(s) are not significantly different at *p* < 0.05.

**Table 5:** Correlation coefficients among shoot dry weight, nodule number, nodule dry weight, N concentration, N content, and SE in common bean.

Variables	Shoot dry weight	Nodule number	Nodule dry weight	Nitrogen concentration	Nitrogen content	SE
Root dry weight	0.245^*^	0.166	−0.018	0.095	0.175	0.175
Shoot dry weight		0.056	0.096	0.275^*^	0.545^**^	0.546^**^
Nodule number			0.703^**^	−0.032	−0.042	−0.043
Nodule dry weight				−0.106	−0.087	−0.087
Nitrogen concentration					0.948^**^	0.948^**^
Nitrogen content						1.000^**^

^∗,∗∗^Correlation is significant at *p* < 0.05, 0.01, respectively.
